# Evaluation of the Effect of Two Different Systemic Doses of *Viola Odorata *on Prevention of Induced Tongue Dysplasia in Rats

**Published:** 2016-09

**Authors:** Sanaz Helli, Hossein Damghani, Daryoush Mohajeri, Mehran Mesgari Abbasi, Rana Attaran, Maryam Zahed

**Affiliations:** 1 Oral Medicine Specialist, Tabriz University of Medical Sciences, Tabriz, Iran.; 2 Post-graduate Student, Dept. of Oral Pathology, School of Dentistry, Tabriz University of Medical Sciences, Tabriz, Iran.; 3 Dept. of Pathobiology, Tabriz Branch, Islamic Azad University, Tabriz, Iran.; 4 Drug Applied Research Center, Tabriz University of Medical Sciences, Tabriz Iran.; 5 Dept. of Oral and Maxillofacial Medicine, Tabriz University of Medical Sciences, Tabriz, Iran.; 6 Dept. of Oral and Maxillofacial Medicine, Dental Disease Research Center, School of Dentistry, Shiraz University of Medical Sciences, Shiraz, Iran.

**Keywords:** 4-nitroquinoline-1-oxide, Viola Odorata, Tongue, Dysplasia

## Abstract

**Statement of the Problem:**

Oral cancer is among the ten most common cancers worldwide. It affects the life quality of patients in many ways.

**Purpose:**

The aim of this study was to compare the effects of two different systemic doses of *Viola Odorata* syrup on the prevention of 4-Nitroquinoline-1-oxide (4-NQO) induced tongue dysplasia in rats.

**Materials and Method:**

Forty-eight male Wistar rats were divided into four groups of A, B, C and D. Group A served as the control group. The rats in groups B to D received 30 ppm of 4-NQO in drinking water for 12 weeks. Additionally, the rats in groups B and C received *Viola Odorata* syrup at doses of 15 and 5 ml/kg, respectively, 3 times a week. Body weights were measured three times a week. At the end, the rats were euthanized and the tongue was removed. Histological evaluations for carcinogenesis were carried out under a light microscope.

**Results:**

The mean body weight of the rats in groups B, C, and D were lower than that in group A (*p*< 0.01). After 12 weeks of treatment, microscopically no histological changes of the tongue base epithelia were observed in the control group. The rats in group B did not show severe dysplastic changes; only mild to moderate histological changes including hyperplasia and hyperkeratosis were evident. These incidences were significantly more apparent in groups C with moderate to severe changes (*p*< 0.05) and group D with severe dysplastic changes (*p*< 0.01). Almost all rats in group D had hyperplasia and manifested all of the stages of dysplasia.

**Conclusion:**

*Viola Odorata* extract has dose-dependent inhibitory effects on the development of tongue induced dysplasia.

## Introduction

Oral cancer is among the ten most common cancers worldwide with a multi-factorial cause.[1] The 5-year survival rate is directly related to the stage of disease at diagnosis. Therefore early preventive measures and treatment modalities can decrease the incidence and progression of the disease.[2] Moreover, since many complications of oral cancer treatments such as xerostomia and mucositis affect the life quality of patients, finding an adjuvant medication for the improvement of patient condition seems necessary.[3]

Free oxygen radicals such as super oxide, hydroxyl, and hydrogen peroxide are influential in the incidence of malignancies like oral cancer. These oxidative agents cause DNA breakdown and augment the expression of proto-oncogenes and the damage of tumor suppressor genes.[4] One of the predominant elements of oxidative reactions is inflammation. In presence of inflammatory factors, the production of oxidative agents such as nitric oxide and free oxygen radicals are increased.[3]

Antioxidants are agents that destroy the consequence of free radicals in two ways; they can prevent the production of free radicals in the human body, and if produced they can decrease their effects in the body. Antioxidants can improve the function of the immune system and increase the defensive abilities of the body and therefore, decrease the incidence of cancer and infections.[3]

The employed antioxidants are either natural (extracted from animals and plants) or synthetic. In recent years, concerning health issues, great deal of attention has been given to natural antioxidants. Extensive research has been carried out to apply these agents in food products instead of synthetic antioxidants.[5] It is important to note that natural antioxidants do not have the disadvantages of synthetic antioxidants, for example cases of severe bleeding in organs like pancreases has been reported in laboratory animals for synthetic antioxidants. Besides, they can also help maintain the health of individuals when used.[6] Moreover, it is confirmed that the biological nature of traditional and herbal medicine is more compatible with the human body and has fewer side effects. Therefore, currently in many countries around the world, traditional medicine, especially herbal medicine is used for prevention and treatment of diseases.[7]

Iran has a climate rich in plant coverage and owing to this fact, antioxidants with a natural source have been given much attention. One of these agents that are being produced in Iran is Odorata syrup extracted from the plant *Viola Odorata*.[8] The mechanism of action of this agent is to damage and break down the cell wall of cancerous and anti-inflammatory cells. Many laboratory (animal and cellular) and clinical studies have been conducted on this agent that confirm the anticancerous effects of Odorata. Improvement of life quality in patients with malignancies and positive outcomes with no side effects are other benefits verified for this medicinal plant.[8-10]

Recent studies revealed the dominant effects of cyclotides of *Viola Odorata* as anticancer agents on gastric, intestinal, rectal, breast, uterine, joints, and pharyngeal cancer. It is reported that its conjunction with chemotherapy can increase the survival rate of patients.[9] Additionally, in cases that chemotherapy is contraindicated, this agent can be used as an alternative with no special care.[9-10]

The indication of this agent according to the studies on cancerous cells is malignancies of the hepatic and bile ducts, intestinal, and breast cancers. All studies give credence to the positive effects of Odorata on the inhibition of the cell cycle in cancerous cells. Moreover, a fairly good toleration by the patients and least interference with the function of different organs are its main privileges.[11-12]

The effects of *Viola Odorata* extract on oral cancer have not been evaluated so far. Since many oral cancers arise from precancerous lesions, the aim of this research is to evaluate the effects of this drug on precancerous lesions of the oral cavity. Furthermore, the effective anticancer doses of the drug used in this study have not been previously determined. In fact, this study evaluated the effectiveness of two different systemic doses of Odorata syrup on induced tongue dysplasia in Wistar rats.

## Materials and Method

**Animals**

Forty eight adult male Wistar rats (10 weeks old) with an average weight of 220 g were obtained from the Animal Lab of Tabriz University of Medical Sciences. After quarantining and acclimatizing the animals to the laboratory conditions for 2 weeks, each rat was housed in a metal cage with a hardwood chip for bedding, in an air-conditioned room under 12-h light/12-h dark cycles and at a temperature of 22±2°C for the course of the study. 

**Chemicals**

4-Nitroquinoline-1-oxide (4-NQO) was purchased from Sigma Inc. (Germany). 4-NQO solution (30 ppm) was prepared twice weekly by dissolving the carcinogenic agent in distilled water and was given to the rats in light-opaque covered bottles. The rats had access to this water ad libitum, during the experiment. *Viola Odorata* syrup was prepared by Goldaru Inc. (Isfahan; Iran).

**Methods**

The Wistar rats were randomly allocated into four groups (A, B, C, D) of 12 animals each. Group A served as control and was given the basal diet and tap water without 4-NQO. The rats in Groups B to D received 30 ppm 4-NQO in drinking water for 12 weeks. When the feeding of 4-NQO was started the rats of groups B and C, received *Viola Odorata *extract at new comparing doses of 15 and 5 ml/kg, respectively, 3 times per week through gavage. Some rats died during the experiment. All the procedures complied with the animal care protocols and regulations observed by the Animal Care and Use Committee of Tabriz University of Medical Sciences.

At the end of the experiment, mean body weights of the rats were calculated to evaluate the potential toxicities. The animals were euthanized and all the tongue tissue was removed. The collected specimens were fixed in 10% buffered formalin and embedded in paraffin. Then, a 5-μm thick microscopic section was prepared for each rat through hematoxylin-eosin staining method. Histological evaluations for carcinogenesis were performed. Epithelial lesions of the tongue were diagnosed according to the criteria described by Bánóczy and Csiba[13] and Kramer *et al.*[14]

**Statistical analysis**

The Statistical Package for Social Sciences (SPSS Inc.; Chicago, IL, USA), version 17.0, was used for statistical analysis. All quantitative data are presented as mean ± standard deviation (SD). Statistical analysis on the incidence of lesions was performed using Fisher's exact probability test or Chi-square test. The data including body weight were compared by ANOVA test followed by Duncan’s post-hoc test. The results were considered statistically significant if the P value was 0.05 or less. 

## Results

**Figure 1:**
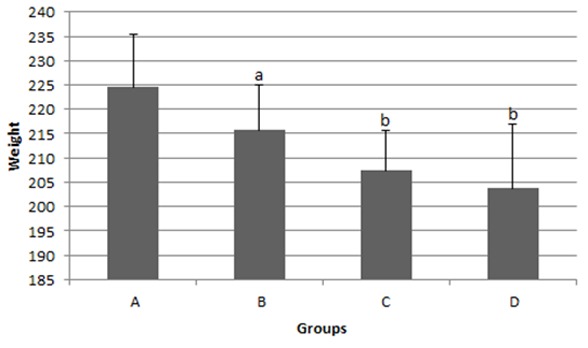
Comparison of the effect of *Viola Odorata* extract on body weight among the experimental groups (mean±SD), and the p value for each group compared to Group A (*p*< 0.05)a, (*p*< 0.01) b.

In the present study 7 of the experimented rats died during the evaluation; 2 from group B, 2 from group C, and 3 from group D. Only 41 rats were alive till the end of the study. The mean body weights at the end of the study are indicated in Figure 1. The mean body weight of rats in group B (*p*< 0.05), C and D (*p*< 0.01) were significantly lower than that of group A. There were no significant differences between the groups C and D from this point of view.

**Figure 2:**
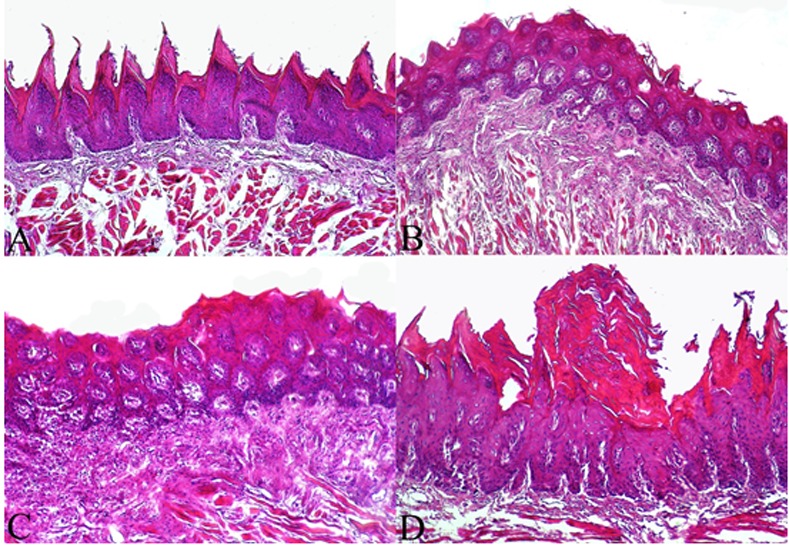
Histological appearance of the tongue epithelium of the rats after 12 weeks of experiment (H&E staining, Original magnification ×40). a: (control group) Normal tongue with no histological changes. b: (4-NQO+15 ml/kg* Viola Odorata* extract) Mild hyperplasia and hyperkeratosis. c: (4-NQO+5 ml/kg *Viola Odorata* extract) Moderate hyperplasia and hyperkeratosis. d: (4-NQO) Severe hyperplasia and hyperkeratosis.

After 12 weeks of treatment in the present study, hyperplasia and three stages of dysplasia (mild, moderate and severe dysplasia), that are considered to be pre-neoplastic lesions for oral cancer, were present in the tongue of the rats in all groups, except group A. The tongues in group B did not show severe dysplastic changes; meanwhile, hyperplasia without atypia and mild to moderate dysplastic changes were detected in this group. These incidences were significantly less than those seen in groups C (*p*< 0.05) and D (*p*< 0.01). However, only one rat from group B (given 15 ml/kg of *Viola Odorata *extract during 4-NQO administration) had moderate dysplasia and one rat from group C (given 5 ml/kg of *Viola Odorata* extract during 4-NQO administration) had severe dysplastic changes. Almost all rats in group D had hyperplasia and manifested all stages of dysplasia. The incidences of such lesions are listed in tables 1 and 2. Microscopically, no histological changes of the tongue base epithelia were observed in the control group (Figure 2a).

**Table 1:** Incidence of tongue pre-neoplastic changes of rats given 4-NQO together with *Viola Odorata* extract

**Group**	**Treatment**	**Number of rats examined**	**Number of rats with**		
			**Normal**	**Hyperplasia**	**Dysplasia**
A	Control	12	12/12^a^	0/12^a^	0/12^a^
B	4-NQO+Viola Odorata extract (15ml/kg)	10	4/10^b^	6/10^b^	3/10^b^
C	4-NQO+Viola Odorata extract (5ml/kg)	10	2/10^c^	8/10^c^	6/10^c^
D	NQO alone	9	0/9	9/9	8/9

Different superscript show signiﬁcant differences from group D by Fisher’s exact probability test (^a^*p*<0.001, ^b^*p*<0.01 and ^c^*p*<0.05).

**Table 2:** Incidence of tongue dysplasia in each group of rats

**Group**	**Treatment**	**Number of rats** **with dysplasia**	**Number of rats**		
			**Mild dysplasia**	**Moderate dysplasia**	**Severe dysplasia**
A	Control	0/12	0/12^a^	0/12^a^	0/12^a^
B	4-NQO+Viola Odorata extract (15ml/kg)	3/10	2/10^b^	1/10^b^	0/10^b^
C	4-NQO+Viola Odorata extract (5ml/kg)	5/10	3/10^c^	2/10^c^	1/10^b^
D	NQO alone	8/9	2/9	3/9	3/9

Different superscript show signiﬁcant differences from group D by Fisher’s exact probability test (^a^*p*<0.001, ^b^*p*<0.01 and ^c^*p*<0.05).

Mild to moderate histological changes including hyperplasia and hyperkeratosis with thickened spinous cell layer was evident after 12 weeks of treatment in the tongue base epithelia of group B (4-NQO + 15 ml/kg of* Viola Odorata *extract). In this group, tongue base epithelial dysplasia was also found only in mild to moderate forms (Figure 2b).

Besides moderate to severe hyperplasia and hyperkeratosis seen throughout the whole thickness of the tongue epithelia, moderate to severe dysplasia of tongue base epithelia was found in group C (4-NQO+ 5 ml/kg of* Viola Odorata *extract) (Figure 2c).

**Figure 3:**
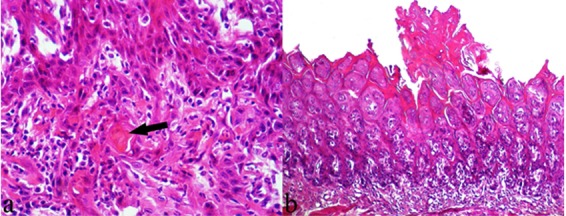
Histological appearance of the tongue epithelium of the rats, 12 weeks after starting the treatment with 4-NQO (Group D) (H&E staining). a: Loss of polarity of epithelium and development of a number of small cell nests in spinous layer with primary keratin pearl formation (arrow) (Original magnification ×250). b: Development of exophytic hyperplasia and hyperkeratosis in epithelial tissue of the tongue tip (Original magnification ×10).

In addition to severe epithelial hyperplasia of tongue, evidence of severe dysplasia (a pre-neoplastic change) in the tongue base epithelia was identified in group D (4-NQO alone) (Figure 2d). Atypia and loss of polarity of epithelium, as well as development of a number of small cell nests in the spinous layer with primary keratin pearl formation were found in rats with severe dysplasia (Figure 3a). Moreover, formation of severe exophytic hyperplasia was found in the epithelial tissue of the tongue tip in group D (Figure 3b).

## Discussion

Chemotherapy, as a treatment of advanced cancer stages, can have an impact on the quality of life of patients and is a major cause of morbidity in this group.[1] Attempts are now under way to improve the side effects of anticancer treatments with the help of traditional and adjunctive medicine.[15-19] Many traditional healing plants have been shown to be well tolerated in humans after several hundred years of empirical testing against specific diseases.[15, 20] Apart from their traditional applications, these plants are now being tested for their effects on diseases that are more widespread, like certain neoplasm.[15-19] Applying traditional medicine in oncology is to add adjunctive treatment modalities to aggressive customary treatments and therefore reduce the destructive effects of usual cancer therapies.[15]

Many organic substances have been examined for their antitumor effects in oral cancer.[16-19]

The sweet violet or *Viola Odorata* is available in Iran in form of 10% concentrated syrup, extracted from the plant. It is used as an adjunctive medicine in gastrointestinal cancer therapies and also for the prevention of metastasis after tumor surgeries. In addition, it has been traditionally applied for the treatment of acute and chronic bronchitis, asthma and flu symptoms. This plant consists of essence, alkaloid, glycoside, salicylic acid, methyl ester, beta-nitro-propionic acid, vitamin C, saponins, and polin.[8, 20] The most effective component of the *Viola Odorata *is a group of peptides named cyclotides.[11] Cycloviolacin O2 (CyO2), a cyclotide derived from *Viola Odorata* was found to have antitumor effects and can cause cell death by membrane permeabilization. Interestingly, CyO2 does not produce significant membrane disruption in primary human brain endothelial cells; this suggests that cyclotides are specified to induce pore formation only in highly proliferating tumor cells.[9, 11]

Our study showed that *Viola Odorata* extract has potential anticancer effects in tongue dysplasia of rats. The anticancer effects of this plant derivative have been investigated,[9, 21] but no previous reports exist on the effects of *Viola Odorata* on oral cancer.

In the present study, the effect of *Viola Odorata* on the prevention of tongue dysplasia in rat was evaluated by monitoring the weight changes and histopathologic changes. The results showed a significant weight loss in the groups receiving the plant derivative and 4-NQO compared to the negative control group with no medications. On the other hand, there were no significant differences in weight loss between the two groups that were administered with different doses of *Viola Odorata*. It was consistent with the results we obtained in our previous study.[19] In addition, Sohrabi *et al.* found that the carcinogenic medicine 4-NQO resulted in weight loss and pathologic changes.[22] Interestingly, there was a recent report of weight loss with the use of the crude extracts of the leaves of *Viola Odorata*. Siddiqi *et al.* reported that this plant exhibited blood pressure-lowering effects in rats under anesthesia and reduced body weight and antidyslipidemic effects which might be due to the inhibition of synthesis and absorption of lipids, as well as antioxidant activities.[23] It can be concluded that, in general, weight loss is induced by both 4-NQO and *Viola Odorata*; in other words, when this plant derivative is applied in cancer treatments, weight loss should be considered as an undesirable side effect besides the beneficial therapeutic effects. But more precise evaluations of the reason behind weight loss are necessary in future studies.

The antitumor effects of *Viola Odorata* and its cyclotides have been investigated *in vitro* and *in vivo*. In Gerlach *et al.*'s *in vitro* study in 2010, a conclusion was drawn that *Viola Odorata* has antitumor effects and causes cell death by membrane permeabilization in breast cancer line. Studies using fluorescence microscopy demonstrated increased cellular internalization of doxorubicin, a chemotherapeutic drug, in drug resistant cells when co-exposed to CyO2, the cyclotide of *Viola Odorata*.[9] Another paper was published in the same year reporting the antioxidant and free radical scavenging results of this medication, whilst employing six various established *in vitro* systems.[10]

Likewise, Burman *et al.* conducted *in vivo* studies on toxicity and antitumor activity of CyO2 in mice. Surprisingly, they noted that despite the *in vitro* sensitivity, no significant antitumor effects were detected *in vivo* for this cyclotide, neither with single dosing nor with repeated dosing. In summary, they indicated that antitumor effects were minor or absent at tolerable (sublethal) doses, besides that CyO2 had a very abrupt *in vivo* toxicity profile, with lethality after a single injection of 2 mg/kg, but with no signs of discomfort in animals at 1.5 mg/kg. Repeated dosing of 1 mg/kg gave a local-inflammatory reaction at the site of injection after 2–3 days; lower doses revealed no complications.[21] It was different from our study most probably due to the fact that a plant derivative has many components and the favorable outcomes are not related to one single cyclotide which was tested in Burman *et al.*'s investigation. As can be seen in 2009, Gridling *et al.* reported that the dichloromethane extract of *Pluchea Odorata *exhibited strong anti-proliferative and pro-apoptotic potential *in vitro*. They found this most likely due to the down-regulation of cyclin D1 and acetylation of α-tubulin, respectively. They noted a variety of similar signaling pathways which were commonly upregulated both in inflammatory conditions and in cancer. Dose-dependently, *Pluchea Odorata *arrested the cell cycle in G2-M phase and also triggered apoptosis.[15]

As previously mentioned, the effective therapeutic doses of *Viola Odorata* syrup have not been reported in literature, so far. Similar doses were reported for syrups of other substances in studies on animals.[24-25] Yet, this is the first study that considers two different dosages for the syrup of this special plant. As the results revealed, higher doses (15 ml/kg compared to 5 ml/kg) are more effective in disrupting cancer cells with no evidence of unfavorable side effects. This finding can be in support of future studies that evaluate new and higher doses for better and more successful treatment outcomes.

Other organic substances have also been evaluated for their effects in oral cancer. A similar study was undertaken in 2001 to examine the inhibitory effect of garlic (*Allium Sati*v*um*) on 4NQO-induced tongue carcinogenesis in rats. The results suggested that garlic has chemopreventive effects and plays an important role in protecting cells against cytotoxic and carcinogenic chemicals by scavenging reactive oxygen species. Garlic significantly suppressed the incidence of 4NQO-induced tongue cancer when given in the post-initiation phase, and revealed an absence of carcinomas when administered in the initiation phase.[16] Evaluating the effects of a substance in the initiation and post-initiation phases and comparing these states can help understand the preventive outcomes besides the therapeutic effects, which is the ideal expectation. Further studies would better consider this comparison regarding *Viola Odorata*.

The same researchers carried out a similar study in 2000 to assess the oral chemopreventive potential of S-allylcysteine (SAC), a water-soluble constituent of garlic on 7, 12- dimethylbenz [a] anthracene (DMBA)-induced hamster buccal pouch carcinoma, and found similar results.[26] Noting that the carcinogen used to induce cancer in rats is not that of importance.

In contrast, coffee exerted a tumor enhancing effect when administered during oral cancer induced by 7, 12- dimethylbenz[a]anthracene (DMBA). The researchers of this study reconciled the differences in tumor development to the type of coffee used. In addition, the concentration of various compounds in coffee may vary depending on the processing and brewing.[17] This is also true for other organic or plant derivatives. Different concentrations and methods of preparation are always very crucial in the results that are anticipated from a substance.

In the current study, a difference was seen from mild dysplasia to moderate dysplasia in groups receiving 15mg/kg and 5mg/kg of medication, respectively. This confirms the importance of dosing and hoping for preferable effects with higher doses of medication. But, it should be pointed out that all substances have side effects which are more prevalent when used in higher doses. It is clear that more animal studies which compare different doses are necessary in order to determine the optimal dose to prevent oral cancer and evaluate possible side effects, paving the way for human studies. 

## Conclusion

Based on the results of the present animal study, *Viola-Odorata* has dose-dependent inhibitory effects on the development of dysplasia in tongue. The higher the dose administered the greater the effect of dysplastic prevention. Nonetheless, future animal and human studies seems to be necessary to find the most effective dose.
